# The First Pan-American Medical Congress

**Published:** 1892-12

**Authors:** 


BUFFALO MEDICAL AND SURGICAL JOURNAL
A MONTHLY REVIEW OF MEDICINE AND SURGERY.

*EDITORS:*
THOMAS LOTHROP, M. D. - - WM. WARREN POTTER, M. D.
All communications, whether of a literary or business character, should be addressed to the managing editor: 284 FRANKLIN STREET, BUFFALO, N. Y.

VOL. XXXII. DECEMBER, 1892. NO. 5.

### THE FIRST PAN-AMERICAN MEDICAL CONGRESS.

WHEN the proposition was made to organize a medical congress, to be composed of physicians delegated thereto from all the countries of the Western Hemisphere, it was considered by many as a visionary scheme, or one of doubtful propriety. In view of the multiplication of medical congresses, societies, associations, and organizations, local, State, national, and international, we are not surprised that many felt doubtful regarding the new proposition. It required boldness, energy, sagacity, patience, and endurance to construct the machinery which should furnish motive power for such a vast organization, and to pull the throttle that should set it in motion after all its parts had been put together.

When the history of the first Pan-American Medical Congress is written, it will be found that, no matter who else may have contributed to its success, the great responsible head for its conception and organization is its accomplished Secretary-General. When the Committee of Organization met together to choose the President of the Congress, and other officers to coöperate with that official, it almost in one voice uttered forth the name of William Pepper. It would be difficult, in the United States, to find a man better equipped to be its presiding officer, and to furnish inspiration and impetus for the successful prosecution of its work, than the genius that has made the University of Pennsylvania such a center of educational power and erudition as has its learned provost. It is needless to remind the American medical profession that William Pepper never puts his hand to the lever unless the machinery moves, nor does he start it until it is certain to move in the direction of success. He is a man who never looks backward, who is constantly standing on the foremost and in the highest lookout, surveying the whole field and making plans for an advance all along the line. Some of these thoughts have come to us after having examined the Preliminary Announcement of the First Pan-American Medical Congress which has lately come to our table.

We note that the meeting is appointed to be held in Washington, Tuesday, Wednesday, Thursday, and Friday, September 5, 6, 7, and 8, 1893. Some criticism has been passed upon the date of holding the meeting, but it must be remembered that several conditions confronted the committee when called upon to fix the date, and it had to be settled in a way to interfere as little as possible with other medical gatherings to be held during the year 1893. The eleventh International Congress which meets in Rome the latter part of September, must not be allowed to conflict with the American Medical Congress, nor must the several State and National societies be interfered with in their dates. So, too, must the teachers of the colleges be accommodated, who nearly all feel it their duty to be present at the opening of the several medical schools that usually begin their sessions between the middle of September and the first of October. So, taken all in all, it was thought best to fix upon the first Tuesday in September as the period in which to inaugurate this new all-American Medical Congress. The Preliminary Announcement, which is a duodecimo brochure of sixty-four pages, is the most comprehensive, detailed, and admirable announcement of a medical organization that ever before has been published. It ought to be the pride of every American physician that it is possible in this country, where the government does not primarily assume responsibility for such a meeting, to progress it to the stage indicated in this announcement, wholly by voluntary action on the part of the physicians of the United States.

We need not, at this time, call attention to the work that it is proposed to do at the Congress, but, when we remember the recently threatened cholera invasion, it will appear at once important that quarantine and public sanitation should be discussed by representative medical men in the Western Hemisphere, with a view to establishing some uniform system in regard to the prevention of epidemic infectious diseases. This, if we mistake not, will be one of the important functions of the Congress. Only second in importance thereto will be the question of medical education, medical teaching—medical pedagogics, if you please—which will be considered in the section on Medical Pedagogics, to be presided over by Dr. J. Collins Warren.

The importance of prompt registration is urged upon all. The Secretary-General has issued a circular upon the subject, showing the great expense of the preparations for the Congress, which must be met by advanced registration. Any physician who desires to become a member should send the registration fee, $10.00, to Dr. A. M. Owen, Treasurer, Evansville, Ind. The confusion incident to registration on the first day, when so many are pressing their claims at one time, thus will be avoided, and all the publications of the Congress, preliminary and final, will be secured to the subscribers. Hence, let all physicians who desire to participate in the Congress register without delay.

